# Successful Control of Hepatitis B Virus Reactivation following Restart of Ibrutinib in Chronic Lymphocytic Leukaemia

**DOI:** 10.1155/2021/1862446

**Published:** 2021-10-21

**Authors:** Sara Kristina Viberg Tjønnfjord, Eirik Brekka Tjønnfjord, Zbigniew Konopski, Geir Erland Tjønnfjord

**Affiliations:** ^1^Department of Nephrology, Odense University Hospital, Odense Kløvervænget 6, 5000 Odense C, Denmark; ^2^Department of Haematology, Oslo University Hospital, P.O. Box 4950 Nydalen, No. 0424 Oslo, Norway; ^3^Department of Gastroenterology, Oslo University Hospital, P.O. Box 4950 Nydalen, No. 0424 Oslo, Norway; ^4^KG Jebsen Centre for B-Cell Malignancies, Institute of Clinical Medicine, University of Oslo, P.O. Box 4950 Nydalen, No. 0424 Oslo, Norway

## Abstract

Ibrutinib is a targeted therapy drug that blocks the activity of Bruton's tyrosine kinase, and it is an approved treatment for several mature B-cell malignancies including chronic lymphocytic leukaemia (CLL). Side effects include infections, cytopenia, nausea, and diarrhoea. In this report, we describe a case of hepatitis B reactivation in a female CLL patient undergoing treatment with ibrutinib. Diagnosis was confirmed with highly elevated hepatitis B virus DNA and a prior blood sample confirmed previous exposure. Ibrutinib was paused, and antiviral therapy was initiated with prompt clinical improvement. Ibrutinib was reinitiated shortly after clinical improvement. Thus, our case report demonstrates that systematic HBV screening is essential before starting treatment with ibrutinib. We suggest that antiviral prophylaxis is considered for patients at risk of reactivation, and ibrutinib may be continued following HBV reactivation with proper antiviral treatment.

## 1. Introduction

Ibrutinib is a small molecule drug that binds irreversibly to Bruton's tyrosine kinase (BTK), an important molecule in B-cell signalling. It is an orally administrated drug showing efficacy in mantle cell lymphoma (MCL), chronic lymphocytic leukaemia (CLL), and Waldenström's macroglobulinemia and compared to chemo-immunotherapy ibrutinib treatment has a lower risk of infections [1].

In preclinical studies using CLL cells, ibrutinib has been shown to promote apoptosis, inhibit proliferation, and prevent cells from responding to survival stimuli provided by the microenvironment resulting in a decreased expression of Mcl-1. In clinical studies, the activity of ibrutinib has been described to include a rapid reduction in lymphadenopathy accompanied by transient lymphocytosis, indicating that the drug has direct effects on cell homing and migration [2–5].

The efficacy of ibrutinib has been impressing progression free survival at 2 years >85% and 70% and overall survival at 2 years >95% and 65% in both treatment-naïve and relapsed or refractory CLL, respectively [3, 5, 6]. However, adverse effects have been documented. The most common are infections, cytopenias, diarrhoea, and muscle and joint pain (>10% frequency). Less common are nonmelanoma skin cancer, interstitial lung disease, tumour lysis syndrome, atrial fibrillation, and haemorrhage [7].

Reactivation of latent infections, particularly herpes and hepatitis B and C viridae, is well documented during treatment with anti-CD20 antibodies and purine analogues. However, only a few case reports describe tyrosine kinase inhibitor-induced HBV reactivation in patients with CLL, and there are no guidelines on management of a reactivation.

## 2. Case Presentation

A 53-year-old female was diagnosed with CLL in 2009 [8]. She exhibited swelling of both parotic glands in 2002, but she was not diagnosed with Sjögren's syndrome until 2008 following clinical evaluation and biopsy of the parotic glands showing atrophy and chronic infection. Following a whole body CT scan disclosing generalized lymphadenopathy, she was referred for haematological evaluation. CLL was confirmed by flow cytometry, CLL score 5/5 [9]. Molecular analysis revealed that the CLL cells used the IGHV3-33 gene showing 100% homology to germ line.

First line treatment with six courses of fludarabine, cyclophosphamide, and rituximab (FCR) was initiated in 2011 due to progressive disease, Binet stage B. Complete remission with no residual disease detected by flow cytometry was accomplished. She had no medical history suggesting hepatitis, and she had been seen regularly at our hospital between 2002 and 2016 without any sign of liver enzymes being elevated. Serological screening for hepatitis was not performed.

Breast cancer was diagnosed in 2014 and treated by ablation and adjuvant radio-chemotherapy.

In January 2016, she suffered a CLL relapse, Binet stage C disease, and second-line treatment with FC without rituximab due to extreme lymphocytosis was initiated while waiting for the result of cytogenetic and molecular genetic assessment. She received one course of treatment with no effect, and as the genetic analysis showed homozygous deletion of 13*q*14 and heterozygous *TP*53*-*mutation (c.486del p.Tyr163ThrfsTer7), treatment was altered to ibrutinib 420 mg once daily from late February 2016.

In June 2016, she was admitted to the Department of Gastroenterology due to malaise, fever, jaundice, and elevated liver parameters (Table 1). Two months before the hospitalization, she had a kidney stone attack. Diclophenac had been administered for a couple of days due to epigastric and back pain. Obstructive uropathy with septicaemia or biliary obstruction was the initial diagnosis as a CT scan disclosed a kidney stone in the lower calyx of the right kidney with hydronephrosis, and empirical treatment with piperacillin and tazobactam was administered. Septicaemia was not confirmed, and hepatitis was then considered the proper diagnosis. A PCR test showed hepatitis B virus (HBV) DNA 12,000,000 IU/ml confirming HBV hepatitis. A serum sample from June 2009 was analysed which disclosed previous exposure to HBV (HBsAg positive, anti-HBc antibody positive, and anti-HBs antibody negative). No HBV-DNA was analysed at that time. She was unaware of any previous exposure to HBV.

When hepatitis B reactivation was diagnosed, ibrutinib was halted, and tenofovir 245 mg was administered once daily following which her condition rapidly improved (Table 1). Ibrutinib treatment was resumed in August 2016. Monotherapy with tenofovir was started as recommended by our infectious medicine consultant. Resistance rarely develops during monotherapy with tenofovir [10]. As of April 2019, she was doing very well on continuous treatment with ibrutinib and tenofovir, but two months later, she was diagnosed with a CLL relapse. Venetoclax monotherapy was initiated in October 2019, and tenofovir was continued. By May 2020, she experienced a complete haematological remission. However, three months later, there were signs of progression, and she succumbed from septicaemia in July 2021.

## 3. Discussion

Reactivation of viral infections is common in patients treated for haematological malignancies. The incidence and severity of viral reactivation relate to the severity of the immunosuppression. Antiviral prophylaxis has been shown to be effective. Guidelines generally recommend that any patient receiving immunosuppression or cytotoxic chemotherapy should be screened for HBsAg, anti-HBc, and HBV-DNA [10–12]. Hepatitis virus screening is recommended in lymphoproliferative disorders, in acute leukaemia, and in other patients treated with monoclonal anti-CD20 antibodies, anthracyclines, or corticosteroids, and in autologous stem cell transplantation [10].

Treatment can be either prophylactic or preemptive. Patients with positive HBsAg, where chemo-immunotherapy is planned, should be treated with antiviral prophylaxis for up to 12–18 months. HBV reactivation in patients with resolved hepatitis (positive anti-HBc and negative HBsAg) is an issue of relevance, particularly in cases of haematological malignancies. It is suggested that patients receiving high-risk treatment regimens are handled similarly to patients with chronic HBV. However, the best intervention strategy is not yet known for patients in the moderate risk group [10–12]. Ibrutinib is a relatively new drug targeting B-cell signalling approved for MCL and CLL. The Summary of Product Characteristics (SPC) states that HBV reactivation may occur during ibrutinib treatment and recommends serological testing for HBV and HCV to be done before treatment. Our patient's first line chemo-immunotherapy treatment, and later ibrutinib, was initiated years before national recommendations were introduced. The effect of ibrutinib on HBV reactivation has not been extensively addressed in the literature, and there are no guidelines on management of HBV reactivation during treatment with ibrutinib.

There are a few other case reports addressing handling of HBV reactivation. de Jésus Ngoma et al. observed an asymptomatic HBV reactivation with transaminitis and HBV-DNA elevation during ibrutinib treatment in an 80-year-old patient with CLL [13]. No antiviral prophylaxis was provided. It should be noted that slightly elevated HBV-DNA was measured before treatment, and ibrutinib was initiated only one month after chemo-immunotherapy was stopped without any universal prophylaxis. Herishanu et al. described reactivation of HBV in a patient with CLL previously treated with ibrutinib [14]. Ibrutinib was given as second-line treatment for twelve months, before it was unintentionally stopped. A rapid increase in ALT and AST occurred six weeks later together with a rapid elevation of HBV-DNA. Antiviral treatment was initiated, and ibrutinib was resumed together with antiviral therapy after full remission from HBV reactivation. A recent case report describes a CLL patient with occult HBV, who initially was treated with chemo-immunotherapy together with antiviral prophylaxis [15]. Ibrutinib was initiated three years later due to progression, and the patient developed HbeAg positivity and elevated HBV-DNA. Ibrutinib was paused, antiviral prophylaxis was prescribed, ibrutinib was reinitiated after fifteen days, and HBV-DNA became undetectable.

In contrast, Tedeschi et al. described a case series of 7 CLL patients with positive anti-HBc antibodies and undetectable HBV-DNA before ibrutinib treatment [16]. Patients were monitored every three months for reactivation. However, no reactivation events were detected during a follow-up period of 25 months.

More recently, Molica et al. reported on ibrutinib treatment in three patients with CLL and chronic active HBV infection [17]. The patients received antiviral therapy 2–10 months before initiation of treatment with ibrutinib. Ibrutinib could safely be administered for more than 2 years with continuous antiviral therapy. In our patient, like the patients reported by Molica et al., viral DNA could not be detected more than two years after the initiation of tenofovir while they are enjoying complete control of their CLL on continuous ibrutinib treatment. Whether antiviral therapy can be safely stopped in these patients when continuing ibrutinib therapy is elusive.

We chose to stop ibrutinib for eight weeks when HBV reactivation was diagnosed and tenofovir was initiated in our patient. Reactivation of HBV usually occurs after the second and third course of chemotherapy, and the median interval is 4 months [18]. Since the patient only received one course of chemotherapy, and HBV reactivation occurred more than 5 months later, ibrutinib is the possible cause of reactivation. Plasma HBV-DNA had started to decrease when ibrutinib was resumed. Whether the temporary discontinuation of ibrutinib was necessary remains unknown. At the time, the ECIL-5 guideline on management of viral hepatitis in patients with haematological malignancies had no specific recommendations on prophylaxis or treatment of HBV reactivation in patients treated with BTK inhibitors, but tenofovir or entecavir is recommended as first line treatment when indicated [19]. We, like Molica et al., chose tenofovir monotherapy which proved to be effective. To our knowledge, only one retrospective study has addressed the risk of HBV reactivation in this specific patient group. Hammond et al. found a cumulative incidence of 9.5% during ibrutinib treatment [20]. Thus, there is a relative high risk of reactivation, with possibly severe outcome. In patients with a history of HBV infection, prophylactic antiviral therapy is recommended by the German Society of Hematology and Medical Oncology to prevent reactivation during chemo-immunotherapy and should most likely be applied in patients treated with ibrutinib as well. However, we do not consider a history with HBV infection a contraindication for treating with ibrutinib or other signal inhibitors, if proper precautions are taken and antiviral prophylaxis provided in patients at risk of reactivation.

## Figures and Tables

**Table 1:** Time course of blood values obtained before, during, and after reactivation.

	February 16	April 16	May 16	June 16 (admission to hospital)	June 16 (ibrutinib paused, tenofovir prescribed)	June 16	August 16 (ibrutinib reinitiated with tenofovir)	October 16	January 17	January 18
P-AST (U/L)	30	↑40	↑62	↑714	↑618	↑133	28		23	16
P-ALT (U/L)	22	↑63	↑109	↑1553	↑843	↑304	27		18	24
P-ALP (U/L)		80	79	↑186	↑181	↑148	83		75	67
P-BILI (*μ*mol/L)		9	7	↑182	↑232	↑186	12		7	13
P-INR		1.2		↑1.5	↑1.5	↑1.4	1.1			
HBV-DNA (IU/mL)					↑ 12 000 000	↑170 000		↑ 51	<20	<20

Reference ranges: AST (15–35 U/L), ALT (10–45 U/L), ALP (35–105 U/L), bilirubin (5–25 *μ*mol/L), and INR (0,8–1,2).

## Data Availability

The data used to support the findings of this study are available from the corresponding author upon request.

## Additional Points

(i) All patients with CLL planning to start ibrutinib should be screened for HBV, and a double assurance of negative screening should be included in clinical practice before administration of chemotherapy in order to reduce the possibility of a reactivation. (ii) Previous HBV infection is not a contradiction for treatment with ibrutinib, if proper precautions are taken and antiviral prophylaxis provided in patients at risk of reactivation. (iii) There is a need for international guidelines on the management of viral hepatitis in CLL and related disorders specifically addressing treatment with signal pathway inhibitors.
